# High expression of Linc00959 predicts poor prognosis in breast cancer

**DOI:** 10.1186/s12935-019-0748-7

**Published:** 2019-02-20

**Authors:** Weiru Chi, Sheng Huang, Bingqiu Xiu, Qi Zhang, Zhiming Shao, Jiong Wu, Yayun Chi

**Affiliations:** 1 Department of Breast Surgery, Breast Cancer Institute, Fudan University Shanghai Cancer Center, Building 7, No. 270 Dong An Road, Shanghai, 200032 China; 2 Department of Oncology, Shanghai Medical College, Fudan University, Shanghai, China; 3 The 2nd Department of Breast Surgery, Breast Cancer Center of the Third Affiliated Hospital of Kunming Medical University, Tumor Hospital of Yunnan Province, Kunming, China; 4 Collaborative Innovation Center for Cancer Medicine, Shanghai, China

**Keywords:** Linc00959, Breast cancer, Prognosis, Long non-coding RNA

## Abstract

**Background:**

Accumulating studies have focused on the oncogenic roles of the newly identified lncRNAs in human cancers. The aim of this study was to examine the expression pattern of Linc00959 in BC and to evaluate its biological role and clinical significance in prediction of prognosis.

**Methods:**

Expression of Linc00959 was detected in 290 BC tissues by quantitative reverse-transcription polymerase chain reaction (qRT-PCR). We analyzed the relationship between Linc00959 expression and clinic pathological features of BC patients. The correlation was calculated by SPSS software.

**Results:**

Our results revealed that Linc00959 expression was correlated with ER status (p = 0.005), PR status (p = 0.036), Ki67 (p = 0.025) and HER2 status (p = 0.009). The Kaplan–Meier survival curves indicated that the overall survival (OS) (p = 0.022) and relapse-free survival (RFS) (p = 0.002) were significantly poor in high Linc00959 expression BC patients (p = 0.023). Furthermore, the survival analysis by Cox regression showed that Linc00959 served as an independent prognostic marker in breast cancer (p = 0.004).

**Conclusion:**

Our studies indicate that Linc00959 is significantly associated with poor prognosis and may represent a new marker of prognosis in breast cancer.

**Electronic supplementary material:**

The online version of this article (10.1186/s12935-019-0748-7) contains supplementary material, which is available to authorized users.

## Background

Breast cancer is the most common malignant tumor among women worldwide [1]. It has become one of the main causes of harm to women’s health in China, and it is increasing year by year. In 2008, GLOBOCA estimated that breast cancer is the most frequent cancer in Chinese women, with an Age Standardised Rate (ASR) of 21.6 cases per 100,000 women, while in 2015 with an ASR 268.6 cases per 100,000 women [2, 3]. Although the prognosis of breast cancer patients has been greatly improved under multidisciplinary treatment, some patients still has local recurrence and even distant metastasis, eventually leading to death.

Since the completion of the human genome project, it has been found that only about 2% of the human genome encodes proteins, while 98% of transcripts are non-coding RNAs [4]. LncRNA are transcripts of more than 200 nucleotides without the protein coding portraiture [5]. In addition to the fact that they are highly deregulated in tumors, lncRNAs have been found to act as tumor suppressors or oncogenes.

Multiple studies revealed that lncRNA is involved in the initiation and progression of breast cancer. The role of lncRNAs in breast cancer is widely studied. For example, growth arrest-specific transcript 5 (GAS5), has also been shown to be expressed at low levels in breast cancer tissues, GAS5’s overexpression in breast cancer cell lines induced apoptosis and suppressed proliferation [6]. Lnc-BM can prognostic the progression of brain metastasis in breast cancer (BCBM) patients and may be a promising therapeutic target for BCBM [7]. Among them, HOX transcriptional antisense RNA (HOTAIR) gene is most closely related to breast cancer. HOTAIR interacts with Polycomb Repressive Complex 2 (PRC2) and is required for PRC2 occupancy and histone H3 lysine-27 trimethylation of HOXD locus [8]. Conversely, loss of HOTAIR can inhibit cancer invasiveness, particularly in cells that possess excessive PRC2 activity. Related studies have found that breast cancer patient’s tumor tissue and plasma had high expression of HOTAIR, HOTAIR diagnostic efficacy and specificity is high and can be used as a potential diagnostic marker for breast cancer [9]. These findings suggest that lincRNAs are a new class of potential biomarkers and targets for the treatment of cancer.

Based on recent studies, we used the TCGA lncRNome information as a clinical filter [10] to find several lincRNAs candidates that have cancer-associated genomic alterations and might be correlated with patient survival in breast cancer. The cohort from the study are made of 290 breast cancer patients from Fudan University Shanghai Cancer Center. The research scientists investigated linc00959 in the cohort. In this research, we found that Linc00959 was significantly associated with poor prognosis and represented a new marker of prognosis in breast cancer.

## Methods and materials

### Patients’ samples

A total of 290 primary breast cancer samples of stage I to III invasive carcinoma of no special type cases and adjacent non-cancerous tissue (ANCT) were collected randomly at the Department of Breast Surgery in Fudan University Shanghai Cancer Center (FDUSCC, Shanghai, P.R. China). Each case was given a unique identifier and linked to a database containing clinical–pathological data. ANCT were diagnosed by the pathologists through H.E. staining as to confirm it was normal breast tissue. The tumors were assessed according to the WHO classification by two academic pathologists. In addition, the pathological data including ER, PR, HER2, P53 and Ki67 status/expression were assessed and diagnosed by the pathologists based on the ASCO breast cancer guideline. Patient information and tumor pathology are summarized. This study was approved by the Ethical Committee of Fudan University Shanghai Cancer Center for Clinical Research (Reference number: 050432-4-1212B). Written informed consents were obtained from all the patients.

### RNA extraction and quantitative RT-PCR

Total RNA was extracted using TRIzol reagent (Invitrogen). After converting total RNA to cDNA in a reverse transcription (RT) reaction, qPCR was used to quantitate the mRNA expression levels. To detect Linc00959 expression, we used the SYBRGreen method with primers listed below: *Linc00959* forward 5′ CCAGGCTTCCACTGACTCTG 3′ and Linc00959 exon 2 reverse 5′ TGTTGGGAGACTCTGAACGC 3′. GAPDH was used as an internal control. 2^−delta Ct^ values were used to determine their relative expression.

### Statistical analysis

Analyses were performed using SPSS software. Kaplan–Meier survival analysis was also performed using SPSS. Differences with *p*-values < 0.05 are considered significant. Univariate analysis was used in multivariate analysis on the basis of Cox proportional hazards model. Two-sided p-values were calculated and a probability level of 0.05 was chosen for statistical significance.

## Results

### Correlations between Linc00959 expression and clinical characteristics

To identify the clinical relevance of Linc00959 expression in breast cancer, correlation between Linc00959 expression and clinic pathological parameters such as age, ER/PR, vessel invasion, tumor size, lymph node status and TNM stage were examined (Table 1). To assess the correlation of Linc00959 expression with clinicopathologic data, the expression of Linc00959 in tumor tissues were categorized as low or high according to the ROC curve assessment. Of the 290 breast cancer patients, 67 cases were Linc00959 low expression, and the other 223 cases were Linc00959 high expression. Chi square tests are used to determine the cutoff value of the two groups for the study. Linc00959 expression in breast cancer was significantly correlated with ER status (p = 0.005), PR status (p = 0.036), Ki67 (p = 0.025) and HER2 status (p = 0.009). ER/PR positive patients had a relatively low Linc00959 expression level. Moreover, HER2 negative and/or ki67 positive patients tended to have high Linc00959 expression. However, Linc00959 expression in breast cancer had no relationship with other parameters (Table 1).

**Table 1:** Relationship between Linc00959 expression and clinicopathological features in 290 breast cancer patients for IHC detection

Characteristics	Linc00959		Number of patients (%)	p value
	Low n (%)	High n (%)		
Total	67 (23.1)	223 (76.9)	290	
Age				0.448
< 50	59 (20.3)	188 (2.8)	247 (23.1)	
≥ 50	8 (64.8)	35 (12.1)	43 (76.9)	
Tumor size (cm)				0.871
≤ 2 cm	26 (9)	78 (26.9)	104 (35.9)	
> 2, 5 ≤ cm	39 (13.4)	137 (47.2)	176 (60.7)	
> 5 cm	2 (0.6)	8 (2.8)	10 (3.4)	
Node status				0.100
Negative	34 (11.7)	108 (37.2)	142 (49)	
Positive	33 (11.4)	115 (39.7)	148 (51)	
ER status				*0.005*
Negative	20 (6.9)	111 (38.3)	131 (45.2)	
Positive	47 (16.2)	112 (38.6)	159 (54.8)	
PR status				*0.036*
Negative	23 (7.9)	110 (37.9)	133 (45.9)	
Positive	44 (15.2)	113 (39)	157 (54.1)	
HER-2 status				*0.009*
Negative	39 (13.4)	145 (50)	184 (63.4)	
Positive	24 (8.3)	77 (26.6)	101 (36.6)	
Ki67				*0.025*
Negative	39 (13.4)	93 (32.1)	132 (45.5)	
Positive	28 (9.7)	130 (44.8)	158 (54.5)	
Vessel invasion				0.264
Negative	41 (14.1)	118 (40.7)	159 (54.8)	
Positive	26 (9)	105 (36.2)	131 (45.2)	
Subtype				0.923
I	31 (10.7)	54 (18.6)	85 (29.3)	
II	17 (5.9)	58 (20)	75 (25.9)	
III	8 (2.8)	58 (18.4)	66 (21.2)	
IV	11 (3.8)	53 (18.3)	64 (22.1)	
TNM stage				0.055
I	17 (5.9)	47 (16.2)	64 (22.1)	
II	33 (11.4)	114 (39.3)	147 (50.7)	
III	17 (5.9)	61 (21)	78 (26.9)	

*p* value  was displayed with Italic when it was less than 0.05

*ER* estrogen receptor; *HER-2* human epidermal growth factor receptor 2

p is based on Fisher’s exact test

### Association between Linc00959 expression and patient survival

**Fig. 1:**
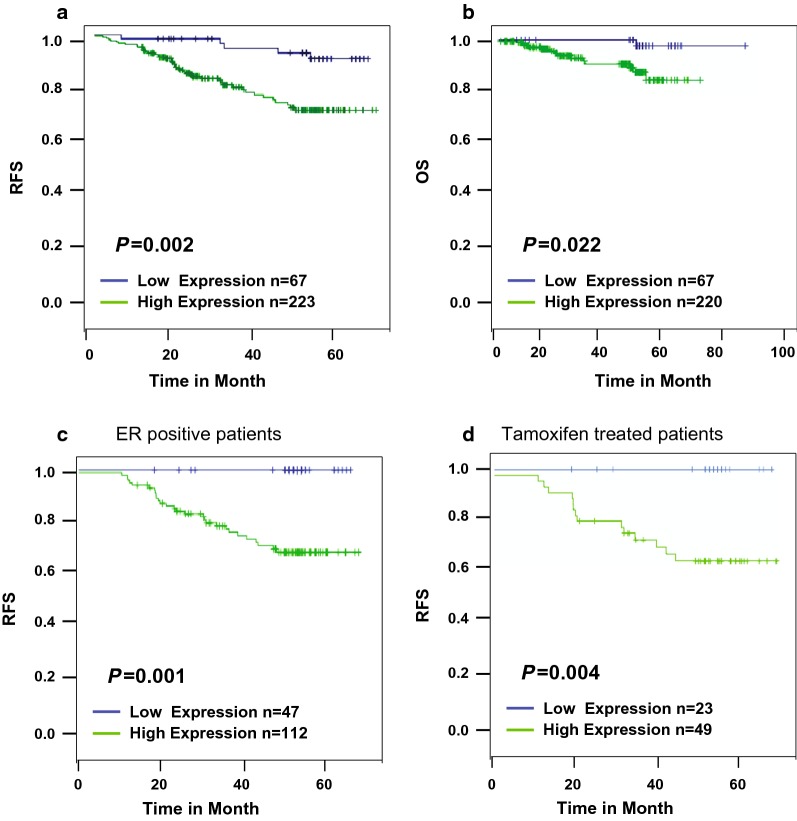
Kaplan–Meier survival curves of breast cancer patients based on Linc00959 expression status (blue lines indicate patients with low Linc00959 expression; green lines indicate patients with high Linc00959 expression). **a** Cumulative disease-free survival curves of breast cancer with high Linc00959 expression displayed a poor RFS (p = 0.002). **b** Cumulative disease-free survival curves of breast cancer patients with high Linc00959 expression displayed a poor OS (p = 0.022). **c** Cumulative disease-free survival curves according to Linc00959 expression status of 159 ER-positive breast cancer patients (p = 0.048). **d** Cumulative disease-free survival curves according to Linc00959 expression status of 192 breast patients treated with tamoxifen (p = 0.034)

All patients were followed up for at least 5 years. OS and RFS curves in low and high Linc00959 expression groups were shown in Fig. 1, and patients with high Linc00959 expression displayed a poor OS (p = 0.022) and RFS (p = 0.002). Further analysis showed that mainly the ER positive patients with high Linc00959 expression displayed worse RFS (p = 0.001), while other types did not (p = 0.865, Additional file 1: Figure S1). ER positive breast cancers dependent on estrogen signaling for proliferation, and the most effective method to stop or slow the growth of these hormone-sensitive tumors is to block estrogen action in the tumor using endocrine therapy. Current endocrine therapies for ER positive breast cancer include: tamoxifen; fulvestrant and aromatase inhibitors (AIs) [11]. As Linc00959 was also associated with ER/PR, we investigated the prognosis of patients treated with tamoxifen (n = 72). Patients with high Linc00959 expression treated with tamoxifen displayed worse RFS (p = 0.004).

Both univariate and adjusted multivariate survival analyses were performed and showed significant differences in RFS between the Linc00959 high and low expression groups. Linc00959 high expression group had a significantly higher incidence of disease events in both univariate analysis (HR = 3.922; 95% CI 1.549–9.927; p = 0.004) (Table 2) and multivariate analysis (HR = 5.411; 95% CI 1.855–15.790; p = 0.002) (Table 3). These data suggest that Linc00959 (p = 0.002), TNM (p = 0.001) and ki67 (p = 0.035) were independent prognostic factors.

**Table 2:** Univariate regression model of prognostic covariates in BC patients

Variable	HR	95.0% CI		p value
		Lower	Upper	
Age (< 50/≥ 50)	1.618	0.828	3.159	0.159
Subtype	1.172	0.910	1.509	0.218
ER (negative/positive)	0.703	0.396	1.250	0.230
PR (negative/positive)	0.807	0.453	1.438	0.467
TNM (I, II, III)	1.325	1.125	1.561	*0.001*
Ki67	1.911	1.075	3.395	*0.027*
Her2 status (negative/positive)	0.712	0.403	1.26	0.244
Vessels invasion (negative/positive)	2.311	1.304	4.097	*0.004*
Tumor size (≤ 2 cm, > 2, 5 ≤ cm, > 5 cm)	1.444	0.865	2.410	0.159
Linc00959 (negative/positive)	3.922	1.549	9.927	*0.004*

*p* value  was displayed with Italic when it was less than 0.05

*CI* confidence interval, *ER status* estrogen receptor status, *PR status* progesterone receptor status, *HER-2* human epidermal growth factor receptor 2, *HR* hazard ratio

**Table 3:** Multivariate regression model of prognostic covariates in BC patients

Variable	HR	95.0% CI		p value
		Lower	Upper	
TNM (I, II, III)	1.500	1.174	1.917	*0.001*
Ki67	1.875	1.044	3.367	*0.035*
Vessels invasion (negative/positive)	2.311	1.304	4.097	0.061
Linc00959 (negative/positive)	5.411	1.855	15.790	*0.002*

*p* value  was displayed with Italic when it was less than 0.05

*CI* confidence interval, *HR* hazard ratio

## Discussion

To our knowledge, Linc00959 was reportedly in non-small-cell lung carcinomas and colorectal cancer [12, 13], but was not previously associated with breast cancer. Sun et al. report that Linc00959 knockdown enhanced colon cancer cell proliferation, invasion, and migration; upregulated N-cadherin and vimentin; and downregulated E-cadherin and Caspase-3. LINC00959 overexpression produced the opposite effects [12]. Linc00959 was reportedly downregulated non-small-cell lung carcinomas [13]. In the future we can use it as reference when we dig in for the molecular mechanism. Colorectal cancer (CRC) patients with high LINC00959 levels had better prognoses than those with low levels, suggesting that LINC00959 may be a useful biomarker for CRC diagnosis [12]. In our study the Kaplan–Meier survival curves indicated that the overall survival (OS) and relapse-free survival (RFS) were significantly poor in high Linc00959 expression BC patients. This suggests that Linc00959 may be an independent prognostic marker in breast cancer.

Breast cancer is the most common malignancy tumor in women. About 70% of patients with breast cancer have tumors presenting ER [14]. However, the development of drug resistance in breast cancer cells is the main cause of treatment failure. Endocrine therapy resistance in breast cancer is a major challenge in the treatment of hormone receptor positive breast cancer. Further explorations of the potential biomarkers to predict patient outcome of Tamoxifen-resistance breast cancer cells and target for therapies are urgently needed. Our data showed that Linc00959 expression was correlated with ER status, PR status, Ki67 and HER2 status. Nowadays, many lncRNA which have shown effective prediction ability. For example, lncRNA HOTAIR is directly repressed by ER and its up-regulation promotes ligand-independent ER activities and contributes to tamoxifen resistance [15]. Tumor-intrinsic plasticity of ERα and H3K27me3 can be a hallmark of endocrine therapy resistance in breast cancer and may ultimately be applicable to guide endocrine treatment selection for patients with breast cancer [16]. Wang et al. [17] developed and validated a novel prognostic tool based on 11-lncRNAs to improve the prediction of disease recurrence for ER-positive BC patients who were treated with tamoxifen as unique endocrine therapy and it can successfully classify patients into high-risk and low-risk groups. The recent active hot spots had focused on lncRNAs can serve as miRNA sponges by competitively binding to miRNA response elements to suppress their expression and function [18]. According Jiang et al. [19] they integrated miRNA–lncRNA signature that can effectively classify TNBC patients into groups with low and high risks of disease recurrence. LncRNA DSCAM-AS1 acts as a competing endogenous RNA of miR-137 and regulates EPS8 to promote cell reproduction and suppresses cell apoptosis in Tamoxifen-resistant breast cancer [20]. As Linc00959 was also associated with ER/PR, we investigated the prognosis of patients treated with tamoxifen. Patients with low Linc00959 expression treated with tamoxifen displayed worse RFS. The results of our study indicate that Linc00959 may represent a new prognostic marker in breast cancer and may be related to endocrine therapy resistance.

The mechanisms accounting for linc00959 associations remain unclear, and further experimental studies were needed to supply the better understanding of linc00959 signature. The next step in-depth study as it may be related to endocrine therapy resistance we can use this as a point cut to investigate thoroughly concrete mechanism of linc00959.

## Conclusion

Our studies first uncover the role of a new lincRNA Linc00959 in breast cancer through investigating its expression in a cohort of breast cancer patients and analyzing its correlation with prognosis. The results show that Linc00959 is significantly associated with poor prognosis, especially in ER positive breast cancer treated with tamoxifen and it may represent a new marker of prognosis in breast cancer.

## Additional file

**Additional file 1: Figure S1.** Cumulative relapse-free survival curves according to Linc00959 expression status of 131 ER-negative breast cancer patients.
