# Supplementary figures and images for: High expression of Linc00959 predicts poor prognosis in breast cancer
Source: Cancer Cell Int. 2019 Feb 20;19:39. doi: 10.1186/s12935-019-0748-7 (PMC6381736; doi:10.1186/s12935-019-0748-7)

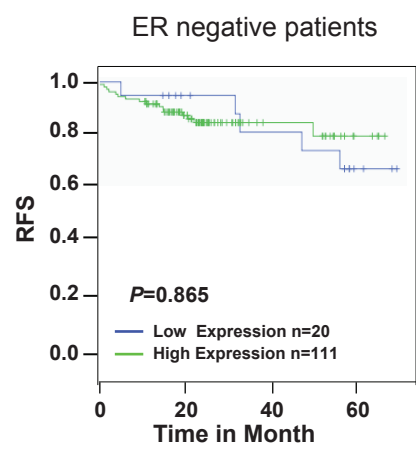

Supplement: Supplementary file 1 — Additional file 1: Figure S1. Cumulative relapse-free survival curves according to Linc00959 expression status of 131 ER-negative breast cancer patients. [file 12935_2019_748_MOESM1_ESM.pdf]
